# Exposure to bacterial products lipopolysaccharide and flagellin and hepatocellular carcinoma: a nested case-control study

**DOI:** 10.1186/s12916-017-0830-8

**Published:** 2017-04-04

**Authors:** Veronika Fedirko, Hao Quang Tran, Andrew T. Gewirtz, Magdalena Stepien, Antonia Trichopoulou, Krasimira Aleksandrova, Anja Olsen, Anne Tjønneland, Kim Overvad, Franck Carbonnel, Marie-Christine Boutron-Ruault, Gianluca Severi, Tilman Kühn, Rudolf Kaaks, Heiner Boeing, Christina Bamia, Pagona Lagiou, Sara Grioni, Salvatore Panico, Domenico Palli, Rosario Tumino, Alessio Naccarati, Petra H. Peeters, H. B. Bueno-de-Mesquita, Elisabete Weiderpass, José María Huerta Castaño, Aurelio Barricarte, María-José Sánchez, Miren Dorronsoro, J. Ramón Quirós, Antonio Agudo, Klas Sjöberg, Bodil Ohlsson, Oskar Hemmingsson, Mårten Werner, Kathryn E. Bradbury, Kay-Tee Khaw, Nick Wareham, Konstantinos K. Tsilidis, Dagfinn Aune, Augustin Scalbert, Isabelle Romieu, Elio Riboli, Mazda Jenab

**Affiliations:** 1 Department of Epidemiology, Rollins School of Public Health, Emory University, Atlanta, GA USA; 2 Winship Cancer Institute, Emory University, Atlanta, GA USA; 3 Center for Inflammation, Immunity, and Infection Institute for Biomedical Sciences, Georgia State University, Atlanta, GA 30303 USA; 4 Section of Nutrition and Metabolism, International Agency for Research on Cancer (IARC-WHO), Lyon, France; 5 Hellenic Health Foundation, 13 Kaisareias Street, Athens, GR-115 27 Greece; 6 Department of Hygiene, Epidemiology and Medical Statistics, WHO Collaborating Center for Nutrition and Health, Unit of Nutritional Epidemiology and Nutrition in Public Health, University of Athens Medical School, Athens, Greece; 7 Department of Epidemiology, German Institute of Human Nutrition Potsdam-Rehbruecke, Nutrition, Immunity and Metabolism Start-up Lab, Nuthetal, Germany; 8 Danish Cancer Society Research Center, Copenhagen, Denmark; 9 Section for Epidemiology, Department of Public Health, Aarhus University, Aarhus, Denmark; 10 Université Paris-Saclay, Université Paris-Sud, UVSQ, CESP, INSERM, Villejuif, France; 11 Gustave Roussy, Villejuif, F-94805 France; 12 Department of Gastroenterology, Assistance Publique-Hôpitaux de Paris (AP-HP), University hospitals Paris-Sud, Site de Bicêtre, Paris Sud University, Paris XI, Le Kremlin Bicêtre, Villejuif, France; 13 Human Genetics Foundation (HuGeF), Torino, Italy; 14 Cancer Council Victoria and University of Melbourne, Melbourne, Australia; 15 Division of Cancer Epidemiology, German Cancer Research Center (DKFZ), Heidelberg, Germany; 16 Department of Epidemiology, German Institute of Human Nutrition Potsdam-Rehbruecke, Nuthetal, Germany; 17 Department of Epidemiology, Harvard School of Public Health, Boston, MA USA; 18 Epidemiology and Prevention Unit Fondazione IRCCS Istituto Nazionale dei Tumori Via Venezian, 1 20133 Milano, Italy; 19 Dipartimento di Medicina Clinica Echirurgia Federico II University, Naples, Italy; 20 Molecular and Nutritional Epidemiology Unit, Cancer Research and Prevention Institute – ISPO, Florence, Italy; 21 Cancer Registry and Histopathology Unit, “Civic -M.P. Arezzo” Hospital, ASP, Ragusa, Italy; 22 Molecular and Genetic Epidemiology Unit, HuGeF, Human Genetics Foundation, Torino, Italy; 23 Department of Epidemiology, Julius Center for Health Sciences and Primary Care, University Medical Center Utrecht, Utrecht, the Netherlands; 24 Department for Determinants of Chronic Diseases (DCD), National Institute for Public Health and the Environment (RIVM), Bilthoven, The Netherlands; 25 Department of Gastroenterology and Hepatology, University Medical Centre, Utrecht, The Netherlands; 26 Department of Epidemiology and Biostatistics, The School of Public Health, Imperial College London, London, UK; 27 Department of Social & Preventive Medicine, Faculty of Medicine, University of Malaya, Kuala Lumpur, Malaysia; 28 Department of Community Medicine, Faculty of Health Sciences, University of Tromsø, The Arctic University of Norway, Tromsø, Norway; 29 Department of Research, Cancer Registry of Norway, Institute of Population-Based Cancer Research, Oslo, Norway; 30 Department of Medical Epidemiology and Biostatistics, Karolinska Institutet, Stockholm, Sweden; 31 Genetic Epidemiology Group, Folkhälsan Research Center, Helsinki, Finland; 32 Department of Epidemiology, Murcia Regional Health Council, IMIB-Arrixaca, Murcia, Spain; 33 CIBER Epidemiología y Salud Pública (CIBERESP), Madrid, Spain; 34 Navarra Public Health Institute, Pamplona, Spain; 35 Navarra Institute for Health Research (IdiSNA), Pamplona, Spain; 36 Escuela Andaluza de Salud Pública. Instituto de Investigación Biosanitaria ibs.GRANADA. Hospitales Universitarios de Granada, Universidad de Granada, Granada, Spain; 37 Basque Regional Health Department, San Sebastian, Spain; 38 Public Health Directorate, Asturias, Spain; 39 Unit of Nutrition, Environment and Cancer, Cancer Epidemiology Research Program, Catalan Institute of Oncology-IDIBELL, L’Hospitalet de Llobregat, Barcelona, Spain; 40 Department of Clinical Sciences, Lund University, Malmö, Sweden; 41 Department of Gastroenterology and Nutrition, Skåne University Hospital, Malmö, Sweden; 42 Department of Clinical Sciences, Division of Internal Medicine, Skåne University Hospital, Malmö, Lund University, Lund, Sweden; 43 Department of Surgical and Perioperative Sciences, Kirurgcentrum, Norrlands Universitetssjukhus, Umeå, Sweden; 44 Department of Medicine Sections for Hepatology and Gastroenterology, Umeå University Hospital, SE-90185 Umeå, Sweden; 45 Cancer Epidemiology Unit, Nuffield Department of Population Health, University of Oxford, Oxford, UK; 46 Clinical Gerontology Unit, University of Cambridge School of Clinical Medicine, Cambridge, UK; 47 MRC Epidemiology Unit, University of Cambridge, Cambridge, UK; 48 Department of Hygiene and Epidemiology, University of Ioannina School of Medicine, Ioannina, Greece; 49 Department of Epidemiology and Biostatistics, School of Public Health, Imperial College London, London, UK

**Keywords:** Hepatocellular carcinoma, Lipopolysaccharide, Flagellin, Endotoxins, Prospective studies

## Abstract

**Background:**

Leakage of bacterial products across the gut barrier may play a role in liver diseases which often precede the development of liver cancer. However, human studies, particularly from prospective settings, are lacking.

**Methods:**

We used a case-control study design nested within a large prospective cohort to assess the association between circulating levels of anti-lipopolysaccharide (LPS) and anti-flagellin immunoglobulin A (IgA) and G (IgG) (reflecting long-term exposures to LPS and flagellin, respectively) and risk of hepatocellular carcinoma. A total of 139 men and women diagnosed with hepatocellular carcinoma between 1992 and 2010 were matched to 139 control subjects. Multivariable rate ratios (RRs), including adjustment for potential confounders, hepatitis B/C positivity, and degree of liver dysfunction, were calculated with conditional logistic regression.

**Results:**

Antibody response to LPS and flagellin was associated with a statistically significant increase in the risk of hepatocellular carcinoma (highest vs. lowest quartile: RR = 11.76, 95% confidence interval = 1.70–81.40; *P*_trend_ = 0.021). This finding did not vary substantially by time from enrollment to diagnosis, and did not change after adjustment for chronic infection with hepatitis B and C viruses.

**Conclusions:**

These novel findings, based on exposures up to several years prior to diagnosis, support a role for gut-derived bacterial products in hepatocellular carcinoma development. Further study into the role of gut barrier failure and exposure to bacterial products in liver diseases is warranted.

**Electronic supplementary material:**

The online version of this article (doi:10.1186/s12916-017-0830-8) contains supplementary material, which is available to authorized users.

## Background

Hepatocellular carcinoma (HCC) has several established risk factors, namely chronic infection with hepatitis B and/or C viruses (HBV/HCV), aflatoxin exposure, diabetes, obesity, smoking, and high alcohol consumption [1]. Recent observations from the European Prospective Investigation into Cancer and Nutrition (EPIC) cohort also show a role for dietary and lifestyle exposures in HCC development [2–6] and indicate metabolic differences between cases and controls [7–9]. Other observations suggest that many of these same factors can weaken the colonic epithelial barrier function [10–12], allowing the translocation of toxic bacterial products such as lipopolysaccharide (LPS; also known as endotoxin, an integral part of the outer membrane of Gram-negative bacterial cell walls) and flagellin (the primary structural component of flagella). Overabundance of bacterial LPS from the gut microbiota may trigger chronic inflammation and higher oxidative stress [13]. Since these bacterial cell components are transported to the liver through the portal vein, it has been suggested that they drive the development of metabolic and liver diseases. In fact, animal data suggest that exposure to LPS or flagellin can produce liver inflammation, liver injury, or steatohepatitis [14–16], while human data indicate higher circulating LPS in patients with chronic liver diseases predisposing to HCC (non-alcoholic fatty liver disease [NAFLD] and non-alcoholic steatohepatitis [NASH]) [17–24]. However, despite a probable role of gut-derived bacterial products in the pathogenesis and progression of liver disease, no epidemiologic studies to date have investigated the association between biomarkers of LPS and flagellin and risk of HCC. In consideration of these points, in a first study of its kind, we investigate whether prediagnostic serum anti-LPS- and anti-flagellin-specific immunoglobulin A and G (IgA and IgG) levels are associated with HCC risk within EPIC, a large cohort of geographically diverse Western European populations.

## Methods

### Study design

EPIC is a multicenter prospective cohort study designed to investigate the association between lifestyle and environmental factors and cancer incidence. The rationale and study design are described in detail elsewhere [25]. The study subjects were recruited from the general population, except for Utrecht and Florence (women attending breast cancer screening), the Oxford “Health conscious” subcohort (half are vegetarian), and subsamples of the Italian and Spanish cohorts (blood donors). Lifestyle data were collected from approximately 520,000 men and women aged 20–85 years enrolled between 1992 and 2000 in 23 centers throughout 10 European countries. At recruitment, blood samples were collected from most participants and are stored at the International Agency for Research on Cancer (IARC, Lyon, France) in –196 °C liquid nitrogen for all countries except Denmark (–150 °C, nitrogen vapor) and Sweden (–80 °C, freezers).

Approval for this study was obtained from the IARC Ethics Committee (Lyon, France) as well as from participating EPIC centers.

### Follow-up for cancer incidence

Cancer incidence was determined through record linkage with population-based regional cancer registries (Denmark, Italy, the Netherlands, Norway, Spain, Sweden, and the UK; complete up to December 2008) or via a combination of methods (health insurance records, contacts with cancer/pathology registries, active follow-up through study subjects and next of kin; France, Germany, Greece; complete until June 2010).

### The nested case-control study

#### Ascertainment of case patients and selection of controls

HCC was defined as first incident tumor in the liver (C22.0 as per the 10th Revision of the International Statistical Classification of Diseases, Injury and Causes of Death [ICD-10]). For each identified case, the histology, the methods used to diagnose the cancer, and α-fetoprotein levels were reviewed to exclude metastatic cases or other types of liver cancers as described previously [2]. During the period between recruitment and 2010, a total of 204 HCC cases were identified. Sixty-five cases had no available serum samples (including 21 cases diagnosed after 2006 in Malmö, Sweden, and Denmark and excluded for administrative reasons and lack of biosample availability) and were not included in the analysis; however, they did not differ by lifestyle and demographic characteristics from cases with available serum samples. For each case, one control was selected by incidence density sampling from all cohort members alive and free of cancer (except non-melanoma skin cancer), and matched by age at blood collection (±1 year), sex, center, date (±2 months)/hour (±3 h) of blood collection, fasting status at blood collection (<3/3–6/>6 h); additionally among women, menopausal status (pre-/peri-/postmenopausal) and hormone replacement therapy use at blood collection (yes/no). The final sample size included 139 HCC cases and 139 matched controls.

### Laboratory biomarker measures for serum anti-LPS- and anti-flagellin-specific Ig levels

Serum anti-LPS- and anti-flagellin-specific IgA and IgG levels were quantitated by enzyme-linked immunosorbent assay (ELISA) at Georgia State University (Atlanta, GA, USA), as previously described [26–28]. Briefly, ELISA plates (Costar™) were coated overnight with purified laboratory-made flagellin (100 ng/well; prepared from *Salmonella typhimurium*, strain SL 3201 fljB^-/-^ as previously described [29]) or purified *Escherichia coli* LPS (2 μg/well; from *E. coli* 0128: B12, Sigma, Catalog No. 2887) in 9.6 pH bicarbonate buffer. Serum samples from cases and controls diluted 1:200 were applied to wells coated with flagellin or LPS. After incubation and washing, the wells were incubated either with anti-IgG coupled to horseradish peroxidase (GE, Catalog No. 375112) or, in the case of IgA-specific antibodies, with horseradish peroxidase-conjugated anti-IgA (KPL, Catalog No. 14-10-01). Using the established platform, specificity of anti-flagellin/LPS Igs is observed when the signal is extremely low when using serum from germ-free mice and completely abolished using serum from RAG-1 knockout mice and germ-free mice on an elemental diet. The specificity of the anti-human IgA and anti-human IgG is in accordance with manufacturer's specifications. Quantitation of total immunoglobulins was performed using the colorimetric peroxidase substrate tetramethylbenzidine (TMB), and the optical density (OD) was read at 450 nm and 540 nm (the difference was taken to compensate for optical interference from the plate) with an ELISA plate reader. Data are reported as OD corrected by subtracting background (determined by readings in blank samples) and are normalized to each plate’s control sample, which was prepared in bulk, aliquoted, frozen, and thawed daily as used. Standardization was performed using preparations of known concentrations of IgA and IgG. Matched case-control pairs were handled identically and assayed in the same batch in a blinded fashion. A very low coefficient of variation (CV <5%) between duplicates based on previous assays [30] permitted singleton sample analysis. Based on three positive control samples included in each plate, mean inter-assay CVs were 2.2%, 2.5%, 3.4%, and 4.8% for anti-LPS IgG, anti-flagellin IgA, anti-LPS IgA, and anti-flagellin IgG, respectively. The between-batch CVs were 9.3%, 12.7%, 16.2%, and 11.3% for anti-flagellin IgA, anti-flagellin IgG, anti-LPS IgA, and anti-LPS IgG, respectively.

### Laboratory assays of HBV/HCV status, biomarkers of liver injury, and hsCRP

The present analysis included existing biomarker data for the same set of cases and matched controls [2, 7, 9]. For a total of 100 of the HCC cases (those diagnosed before 2006) and their matched controls, existing data were available for HBV/HCV seropositivity (ARCHITECT HBsAg and anti-HCV chemiluminescent microparticle immunoassays; Abbott Diagnostics, France) and biomarkers of hepatic injury (alanine aminotransferase [ALT], aspartate aminotransferase [AST], gamma-glutamyltransferase [GGT], liver-specific alkaline phosphatase [AP], albumin, total bilirubin, and total protein; ARCHITECT c Systems™; Abbott Diagnostics) [2]. We created the liver damage score by summarizing the number of abnormal values for six liver function tests (ALT > 55 U/L, AST > 34 U/L, GGT men >64 U/L, GGT women >36 U/L, AP >150 U/L, albumin <35 g/L, total bilirubin >20.5 μmol/L; cut-points were provided by the laboratory and were based on assay specifications; range from 0 to 6).

Serum amino acids were measured for all 139 cases and 139 matched controls using the Biocrates AbsoluteIDQ p150 mass spectrometry kit (Biocrates Life Science AG, Innsbruck, Austria) on a QTRAP mass spectrometer (IARC, Lyon, France) [9]. Fischer’s ratio was calculated as the molar ratio of branched-chain amino acids (leucine + valine + isoleucine) to aromatic amino acids ([phenylalanine + tyrosine + histidine + tryptophan] or [phenylalanine + tyrosine]) and was used as an indicator of hepatic functional reserve and severity of liver dysfunction [31, 32]. High-sensitivity C-reactive protein (hsCRP) was measured using a high-sensitivity assay on a Turbidimetric Modular system (Roche, Mannheim, Germany) [7].

### Statistical analyses

No transformations were used for all biomarkers because they were normally distributed. Differences in concentrations of biomarkers among the controls by baseline characteristics were examined by analysis of variance. *P* values for tests of trend (for ordinal variables) or of heterogeneity were reported. Four conditional logistic models were used to assess the strengths of association (incidence rate ratio [IRR] as estimated by odds ratio [OR] [33] with 95% confidence interval (CI) and tests for trend): (1) with matching factors only, (2) with adjustment for potential confounders (smoking status [never, former, current], body mass index [continuous], baseline alcohol intake [continuous], coffee intake [continuous], lifetime alcohol drinking pattern [always heavy, periodically heavy, former heavy, never heavy, former light, light, and never drinkers], physical activity [active, moderately active, moderately inactive, inactive], and level of education [none, primary school, secondary school, more than secondary school, not specified]), and (3) with additional adjustment for Fischer’s ratio (molar ratio of branched-chain amino acids [leucine + valine + isoleucine] to aromatic amino acids [phenylalanine + tyrosine + histidine + tryptophan]); inversely related to severity of liver dysfunction, with lower values of the ratio indicating a more severe liver dysfunction [31, 32]. Serum anti-LPS and anti-flagellin immunoglobulin levels were included individually and in the following logical combinations in models as continuous (per unit increase; approximately equal to 1 standard deviation for each individual biomarker) and as categorical variables, with quartile cut-points based on the distribution in the control subjects: (1) total anti-LPS = anti-LPS IgG + anti-LPS IgA (total exposure to LPS); (2) total anti-flagellin = anti-flagellin IgG + anti-flagellin IgA (total exposure to flagellin); (3) anti-LPS and anti-flagellin IgG = anti-LPS IgG + anti-flagellin IgG (all IgGs, indicating possible systemic response to endotoxins [34]); (4) anti-LPS and anti-flagellin IgA = anti-LPS IgA + anti-flagellin IgA (all IgAs, indicating possible mucosal response to endotoxins [34]); (5) anti-LPS flagellin = anti-LPS IgG + anti-flagellin IgG + anti-LPS IgA + anti-flagellin IgA (total exposure to LPS and flagellin). To test dose responses, trend variables were assigned the median values for each quartile of biomarker.

To partly control for potential pre-existing liver dysfunction, in the multivariable model, we also performed additional adjustment for and stratification by HBV/HCV status and “liver damage score” by summarizing the number of abnormal values for six liver function tests (categorized as 0 = no liver injury, 1–2 = possible minor injury, ≥3 = possible injury; see Additional file 1: Table S1 and Table 4 footnote).

Effect modification on the multiplicative scale for potential biologically plausible effect modifying variables (sex, age at diagnosis, body mass index [BMI, normal vs. overweight/obese], prevalent type 2 diabetes [yes vs. no; data available for a subset of subjects only], smoking [never vs. former/current], lifetime alcohol drinking pattern [ever heavy vs. light/never]) was tested by including interaction terms formed by the product of modifying variable categories and the value of categories of exposure of interest. The statistical significance of interactions was assessed using likelihood ratio tests based on the models with and without the interaction terms.

All statistical tests were two-sided, and *P* values <0.05 were considered statistically significant. Analyses were conducted using the SAS version 9.2 (SAS Institute, Cary, NC, USA) statistical package.

## Results

### Baseline characteristics of participants

HCC cases were diagnosed, on average, 6 years (standard deviation = 3.4) after blood collection and had a greater proportion of current smokers and a greater prevalence of diabetes than controls (Table 1). The mean serum concentration of total anti-LPS and anti-flagellin Igs was higher in HCC cases vs. controls (8.08 vs. 6.86, *P* < 0.001). No difference in total anti-LPS and anti-flagellin Ig levels by HBV/HCV status was observed for both HCC cases (*P* = 0.379) and controls (*P* = 0.722). The Fischer ratio was lower in HCC cases vs. controls (1.33 vs. 1.53, *P* < 0.001) and, among cases, moderately inversely correlated with total anti-LPS and anti-flagellin Igs (*r* = –0.28, *P* < 0.001). Among cases, having potential liver dysfunction as indicated by a liver damage score value ≥3 was associated with higher levels of total anti-LPS and anti-flagellin Igs among cases (*P* < 0.001 compared to cases with a liver damage score value of 0).

**Table 1:** Baseline characteristics of incident HCC cases and matched control subjects within the European Prospective Investigation into Cancer and Nutrition (EPIC) study from 1992 to 2010

Characteristic	Case subjects (*N* = 139)	Matched control subjects (*N* = 139)^a^	*P value*
Men (%)	70.5	70.5	–^e^
Age at blood collection (y), mean (SD)	60.0 (7.3)	60.0 (7.3)	–^e^
Follow-up from blood collection (y), mean (SD)	6.0 (3.4)	--	–
Smoking status (%)			*0.002*
Never smoker	29.7	43.5	
Former smoker	30.4	36.2	
Current smoker	39.1	19.6	
BMI (kg/m^2^), mean (SD)	28.4 (4.7)	27.3 (4.2)	*0.035*
Physical activity (%)			*0.652*
Inactive	7.3	10.9	
Moderately inactive	36.2	31.2	
Moderately active	46.4	48.6	
Active	10.1	9.4	
No. with prevalent diabetes (%)	13.0	7.3	*0.455*
HBV or HCV positive (%)^b^	35.6	3.0	*<0.001*
HBV positive (%)^b^	16.8	2.0	*<0.001*
HCV positive (%)^b^	21.8	2.0	*<0.001*
Liver damage score (%)^b^			
0	29.0	83.2	*<0.001*
1–2	31.0	16.8	
≥3	40.0	0	
Baseline blood biomarkers, mean (SD)			
Anti-LPS IgG + IgA	4.27 (1.38)	3.64 (1.32)	*<0.001*
Anti-flagellin IgG + IgA	3.81 (1.34)	3.22 (1.17)	*<0.001*
Anti-LPS IgG + anti-flagellin IgG	3.62 (1.31)	3.19 (1.19)	*0.005*
Anti-LPS IgA + anti-flagellin IgA	4.46 (1.57)	3.67 (1.40)	*<0.001*
Anti-LPS IgG + IgA + anti-flagellin IgG + IgA	8.08 (2.59)	6.86 (2.34)	*<0.001*
Fischer's ratio^c^	1.33 (0.26)	1.53 (0.24)	*<0.001*
C-reactive protein (mg/L)^d^	2.8 (3.0)	2.0 (2.2)	*0.006*

From the following recruitment centers, number of HCC cases: Denmark (*N* = 21), Germany (*N* = 31), Greece (*N* = 16), Italy (*N* = 28), Spain (*N* = 11), Sweden (*N* = 13), the Netherlands (*N* = 4), United Kingdom (*N* = 15). No eligible case patients were identified in the cohorts of France and Norway, which include women only

^a^Control subjects had to be alive as of the time of diagnosis of the corresponding case patients and were matched with case patients for study center, sex, age at the time of blood collection (±12 months), date of blood collection (±2 months), and time of day of blood collection (±3 h). Women were further matched by menopausal status (pre-, post-, or perimenopausal) and use of exogenous hormones (oral contraceptives for premenopausal women and hormone replacement therapy for postmenopausal women) at time of blood collection

^b^Available for 100 cases and 100 controls

^c^Calculated as the molar ratio of branched-chain amino acids (leucine, valine, isoleucine) to aromatic amino acids (phenylalanine, tyrosine, histidine, tryptophan), an indicator of hepatic functional reserve and the severity of liver dysfunction. Geometric means (SD)

^d^Geometric means and SDs, available for 100 cases and 100 controls

^e^Matching factor

### Lifestyle and dietary factors associated with anti-LPS and anti-flagellin Igs in controls

Among controls, concentrations of biomarkers did not differ statistically significantly by sex, age at blood collection (Table 2), and other factors (Additional file 1: Table S2). A higher BMI was associated with higher concentrations of anti-LPS Igs (*P* = 0.02), anti-LPS and anti-flagellin IgGs (*P* = 0.02), and total anti-LPS and anti-flagellin (*P* = 0.04). Similar patterns were observed for waist-to-hip ratio, a measure of central adiposity, and CRP, a biomarker of chronic systemic inflammation, although they were not statistically significant.

**Table 2:** Mean (95% CI) anti-LPS and anti-flagellin immunoglobulin levels in controls by sex, age at blood collection, and other baseline characteristics

Characteristic	Anti-LPS IgG + IgA	95% CI		Anti-Flagellin IgG + IgA	95% CI		Anti-LPS IgG + Anti-Flagellin IgG	95% CI		Anti-LPS IgA + Anti-Flagellin IgA	95% CI		Anti-LPS IgG + IgA + Anti-Flagellin IgG + IgA	95% CI	
Sex															
Female	3.78	3.37	4.19	3.18	2.82	3.54	3.37	3.00	3.74	3.59	3.15	4.02	6.96	6.23	7.68
Male	3.58	3.32	3.85	3.24	3.01	3.48	3.11	2.88	3.35	3.71	3.43	3.99	6.82	6.35	7.29
*P* value*	0.425			0.771			0.249			0.639			0.762		
Age at blood collection, years															
≤55	3.64	3.21	4.07	3.11	2.72	3.49	3.26	2.87	3.65	3.48	3.02	3.94	6.75	5.97	7.52
55.1–60	3.40	2.93	3.87	3.24	2.82	3.66	2.99	2.57	3.42	3.64	3.15	4.14	6.64	5.80	7.47
60.1–65	3.56	3.15	3.96	3.13	2.77	3.48	3.10	2.73	3.46	3.58	3.16	4.01	6.68	5.96	7.40
>65	4.01	3.53	4.48	3.48	3.06	3.90	3.44	3.01	3.86	4.05	3.55	4.56	7.49	6.64	8.34
*P* value*	0.218			0.270			0.499			0.131			0.215		
BMI, kg/m^2^															
≤25	3.39	2.98	3.79	3.02	2.65	3.38	3.04	2.68	3.40	3.37	2.93	3.80	6.41	5.68	7.13
25.1–29.9	3.55	3.23	3.87	3.24	2.95	3.52	3.03	2.75	3.32	3.75	3.41	4.09	6.78	6.21	7.35
≥30	4.11	3.67	4.55	3.44	3.04	3.83	3.67	3.28	4.06	3.88	3.41	4.35	7.55	6.76	8.34
*P* value*	0.019			0.123			0.022			0.114			0.036		
Waist-to-hip ratio (WHR)															
<0.92 (M)/<0.77 (W)	3.35	2.98	3.73	3.15	2.81	3.49	2.98	2.64	3.32	3.53	3.12	3.93	6.50	5.83	7.18
0.92–0.97 (M)/0.77–0.84 (W)	3.77	3.41	4.14	3.21	2.89	3.54	3.27	2.93	3.60	3.72	3.33	4.11	6.99	6.33	7.64
>0.97 (M)/>0.84 (W)	3.80	3.40	4.20	3.31	2.96	3.67	3.34	2.98	3.70	3.78	3.35	4.21	7.12	6.40	7.83
*P* value*	0.110			0.510			0.153			0.401			0.221		
CRP, mg/L^‡^															
≤1	3.40	3.03	3.76	3.09	2.75	3.42	3.02	2.69	3.36	3.46	3.06	3.86	6.48	5.82	7.14
1–5	3.68	3.25	4.12	3.39	2.99	3.78	3.27	2.88	3.66	3.80	3.33	4.27	7.07	6.29	7.85
>5	3.95	3.36	4.53	3.33	2.80	3.86	3.17	2.65	3.70	4.10	3.47	4.74	7.28	6.23	8.32
*P* value^a^	0.098			0.326			0.493			0.077			0.156		

*All *P* values are based on a test of linear trend, except *P* values for heterogeneity by sex, smoking status, and lifetime alcohol drinking pattern

^a^Available for 100 controls

### Associations of serum anti-LPS and anti-flagellin Igs with risk of HCC

The associations between LPS and flagellin biomarkers with risk of HCC are presented in Table 3 (for logical combination of biomarkers) and Additional file 1: Table S3 (for individual biomarkers). All analysis models showed a statistically significant positive association between high anti-LPS and anti-flagellin Ig levels and HCC risk (for total anti-LPS and anti-flagellin Igs, highest vs. lowest quartiles, matching factors model: IRR = 8.72, 95% CI: 2.78–27.29; most adjusted multivariable model with Fischer’s ratio: IRR = 11.76, 95% CI: 1.70–81.40, *P*_trend_ = 0.021).

**Table 3:** Incidence rate ratios (IRR) and 95% confidence intervals of hepatocellular carcinoma according to categories of and per 1 unit increase in serum anti-LPS and anti-flagellin immunoglobulin levels, EPIC study, 1992–2010

Biomarker	IRR (95% CI)					OR _per ↑1 unit_
	Q1	Q2	Q3	Q4	*P*_trend_	
Anti-LPS IgG + IgA, *n* case/control	20/35	25/34	35/35	59/35		
Matching factors^a^	Ref.	1.34 (0.61–2.98)	2.74 (1.13–6.65)	11.17 (3.46–36.00)	<0.0001	2.19 (1.59–3.03)
Multivariable^b^	Ref.	1.64 (0.56–4.83)	2.52 (0.76–8.39)	17.16 (3.52–83.50)	0.001	2.58 (1.65–4.03)
+ dietary factors^c^	Ref.	1.65 (0.54–5.04)	2.67 (0.77–9.31)	20.06 (3.88–103.0)	0.001	2.68 (1.69–4.23)
+ Fischer’s ratio^d^	Ref.	2.52 (0.70–9.08)	2.17 (0.55–8.63)	13.65 (1.91–97.80)	0.017	2.05 (1.25–3.36)
Anti-Flagellin IgG + IgA, *n* case/control	22/34	25/36	37/35	55/34		
Matching factors^a^	Ref.	1.09 (0.5–2.38)	2.35 (1.03–5.37)	7.11 (2.46–20.55)	<0.0001	2.18 (1.56–3.06)
Multivariable^b^	Ref.	1.05 (0.37–2.93)	2.87 (1.00–8.20)	7.78 (2.19–27.57)	0.001	2.31 (1.51–3.54)
+ dietary factors^c^	Ref.	1.21 (0.40–3.59)	3.07 (1.05–9.01)	6.72 (1.88–24.02)	0.003	2.25 (1.47–3.47)
+ Fischer’s ratio^d^	Ref.	1.52 (0.43–5.37)	4.37 (1.21–15.84)	3.59 (0.79–16.35)	0.025	1.80 (1.12–2.90)
Anti-LPS IgG + Anti-Flagellin IgG, *n* case/control	24/34	29/36	40/34	46/35		
Matching factors^a^	Ref.	1.23 (0.58–2.63)	2.11 (0.99–4.52)	4.08 (1.43–11.64)	0.006	2.02 (1.43–2.85)
Multivariable^b^	Ref.	1.40 (0.53–3.68)	2.92 (1.06–8.06)	5.80 (1.46–23.02)	0.007	2.47 (1.56–3.91)
+ dietary factors^c^	Ref.	1.43 (0.52–3.92)	3.45 (1.18–10.07)	5.37 (1.31–22.10)	0.007	2.57 (1.60–4.12)
+ Fischer’s ratio^d^	Ref.	1.18 (0.39–3.60)	3.89 (1.12–13.58)	2.71 (0.42–17.29)	0.056	1.97 (1.15–3.36)
Anti-LPS IgA + Anti-Flagellin IgA, *n* case/control	18/35	27/34	27/35	67/35		
Matching factors^a^	Ref.	2.16 (0.82–5.72)	2.23 (0.85–5.87)	8.47 (3.01–23.86)	<0.0001	1.83 (1.43–2.36)
Multivariable^b^	Ref.	4.00 (0.96–16.63)	4.91 (1.15–20.9)	14.22 (3.02–66.90)	0.001	1.99 (1.39–2.86)
+ dietary factors^c^	Ref.	5.34 (1.21–23.68)	4.73 (1.06–21.16)	14.97 (3.04–73.50)	0.001	1.98 (1.37–2.86)
+ Fischer’s ratio^d^	Ref.	2.65 (0.55–12.84)	4.49 (0.94–21.6)	8.40 (1.64–43.12)	0.009	1.65 (1.11–2.45)
Anti-LPS IgG + IgA + Anti-Flagellin IgG + IgA, *n* case/control	23/34	22/35	37/35	57/35		
Matching factors^a^	Ref.	0.97 (0.46–2.08)	2.25 (1.03–4.91)	8.72 (2.78–27.29)	<.00001	1.57 (1.31–1.88)
Multivariable^b^	Ref.	1.54 (0.59–4.02)	2.78 (0.94–8.21)	14.01 (2.99–65.60)	0.001	1.72 (1.34–2.21)
+ dietary factors^c^	Ref.	1.71 (0.63–4.68)	2.78 (0.92–8.35)	13.31 (2.78–63.70)	0.002	1.71 (1.33–2.21)
+ Fischer’s ratio^d^	Ref.	1.75 (0.58–5.27)	2.19 (0.63–7.66)	11.76 (1.70–81.40)	0.021	1.48 (1.13–1.94)

^a^IRRs and 95% confidence intervals were estimated by conditional logistic regression conditioned on the matching factors

^b^Base model further adjusted for smoking status (never, former, current), body mass index (continuous), baseline alcohol intake (continuous), coffee intake (continuous), lifetime alcohol drinking pattern (always heavy, periodically heavy, former heavy, never heavy, former light, light, and never drinker), physical activity (active, moderately active, moderately inactive, inactive), and level of education (none, primary school, secondary school, more than secondary school, not specified)

^c^Multivariable model + baseline dietary fiber (g/day), fish and seafood products (g/day), and total energy (kcal/day)

^d^Multivariable model + Fischer ratio calculated as the molar ratio of branched-chain amino acids (leucine, valine, isoleucine) to aromatic amino acids (phenylalanine, tyrosine, histidine, tryptophan), an indicator of hepatic functional reserve and the severity of liver dysfunction

### Effect modifications and sensitivity analyses

For all variables tested, no statistically significant effect modification were observed (all *P* >0.26), except for sex, which demonstrated as borderline non-significant (Table 4; *P* values for interaction by sex ≥0.03, see the footnotes). However, the number of women in the study was much smaller compared to the number of men. We also checked the consistency of our results after the exclusion of the cases diagnosed during the first 2 and 4 years of follow-up to exclude possible reverse causation, since the participants might have modified their diet and/or lifestyle before enrollment due to prediagnostic symptoms. The estimates did not change considerably after these exclusions or in analyses stratified by follow-up time. The magnitude of the effect estimates did not change substantially after excluding persons with positive HBV/HCV status (data not shown) or by further adjustment for HBV/HCV status and liver damage score (Table 4).

**Table 4:** Incidence rate ratios (IRRs) and 95% confidence intervals (in parentheses) of hepatocellular carcinoma per 1 unit increase in serum anti-LPS and anti-flagellin immunoglobulins from analyses stratified according to selected characteristics and sensitivity analyses, EPIC study, 1992–2010

Model	Cases	Anti-LPS IgG + IgA	Anti-flagellin IgG + IgA	Anti-LPS IgG + anti-flagellin IgG	Anti-LPS IgA + anti-flagellin IgA	Anti-LPS IgG + IgA + anti-flagellin IgG + IgA
Stratified analyses						
By sex						
Men	98	5.12 (2.09–12.55)	5.58 (2.11–14.78)	4.93 (2.06–11.79)	2.34 (1.38–3.97)	2.65 (1.58–4.45)
Women	41	1.61 (0.58–4.44)	1.54 (0.54–4.36)	1.21 (0.43–3.41)	1.71 (0.72–4.03)	1.34 (0.75–2.41)
*P*_interaction by sex_		0.062	0.059	0.030	0.402	0.055
Cases diagnosed						
>2 years since blood collection	117	2.75 (1.62–4.67)	2.04 (1.29–3.21)	2.35 (1.38–4.01)	2.05 (1.33–3.15)	1.68 (1.27–2.23)
>4 years since blood collection	97	4.87 (2.18–10.89)	2.52 (1.4–4.54)	3.70 (1.64–8.34)	2.52 (1.47–4.33)	2.11 (1.41–3.15)
By follow-up time^a^						
< 6 y since blood collection	65	2.45 (1.41–4.26)	2.17 (1.27–3.73)	2.43 (1.36–4.37)	1.89 (1.20–2.95)	1.67 (1.22–2.29)
≥ 6 y since blood collection	74	3.16 (1.65–6.06)	2.57 (1.42–4.64)	2.56 (1.38–4.77)	2.23 (1.36–3.63)	1.82 (1.30–2.56)
Sensitivity analyses						
All data, subset with data on HBV/HCV status	100	2.83 (1.61–4.99)	2.62 (1.43–4.81)	2.88 (1.61–5.15)	1.95 (1.24–3.06)	1.83 (1.32–2.55)
Additionally adjusted for						
Liver damage score^b^	100	2.92 (1.02–8.40)	1.76 (0.83–3.72)	2.86 (1.03–7.92)	1.41 (0.75–2.64)	1.56 (0.96–2.51)
HBV/HCV status and liver damage score^b^	100	3.35 (0.82–13.77)	1.66 (0.62–4.44)	2.35 (0.81–6.79)	1.33 (0.64–2.77)	1.60 (0.83–3.06)

IRRs and 95% confidence intervals were estimated by conditional logistic regression conditioned on the matching factors and adjusted for smoking status (never, former, current), body mass index (continuous), baseline alcohol intake (continuous), coffee intake (continuous), lifetime alcohol drinking pattern (always heavy, periodically heavy, former heavy, never heavy, former light, light, and never drinker), physical activity (active, moderately active, moderately inactive, inactive), and level of education (none, primary school, secondary school, more than secondary school, not specified)

^a^Mean follow-up time among cases (6 years) was used as a cut-point

^b^Subjects with liver damage score of 0 and 1 were considered to have normal liver function. Liver damage score ranges from 0 to 6, grouped in categories as 0, 1, ≥2 abnormal liver function tests based on the values provided by the laboratory: ALT >55 U/L, AST >34 U/L, GGT men >64 U/L, GGT women >36 U/L, AP >150 U/L, albumin <35 g/L, total bilirubin >1.2 mg/dL. Available for 100 cases and 100 controls

## Discussion

In this case-control study nested within a large prospective cohort, we observed a statistically significant positive association between prediagnostic serum concentrations of anti-LPS and anti-flagellin Igs and risk of HCC. The strength of the association did not vary substantially by time from enrollment to diagnosis and was only modestly impacted by adjustment for various lifestyle factors and markers reflecting pre-existing liver damage. These findings provide the first prospective epidemiologic evidence on the topic and add to the existing experimental data showing that weakened gut barrier function and subsequent exposure to bacterial products may promote hepatocarcinogenesis [13, 35].

Higher circulating levels of anti-LPS and anti-flagellin Igs are thought to be indicative of chronic exposure to bacterial products LPS and flagellin, which can elicit innate immune and inflammatory responses [36]. High exposure of the liver to these microbial products could be due to their translocation through the disrupted gut barrier, which could be a result of intestinal inflammation, chronic alcohol abuse, early phase hepatic injury, or dietary and/or lifestyle factors [24, 37, 38]. In fact, data from animal studies support an important role for gut microbiota in liver health and disease [13, 39]. Furthermore, patients with NAFLD and NASH, liver diseases that often precede HCC, demonstrate elevated circulating endotoxin levels, LPS, LPS-binding protein, and anti-LPS antibodies [17–24]. It is plausible that dietary and lifestyle factors associated with higher risk of cancer development, such as components of Western-type diets, instigate microbiome changes, favoring the relative abundance of Gram-negative bacteria, and thus leading to increased exposure to LPS. For example, studies in mice show that diets high in fructose or fat can alter gut barrier function, inducing endotoxemia and the development of liver steatosis [11, 40]. Similar observations have been made in humans [21] and for other factors such as obesity [12, 36, 41]. Although there is some debate in the current animal literature as to whether endotoxemia is a cause or consequence of liver damage [24], it is becoming increasingly clear, as noted above, that it is involved in the development and progression of NAFLD [18]. Our results build on this knowledge to show a possible continued role for endotoxemia in HCC development.

The most compelling mechanism for this observation is bacterial dysbiosis (abundance of Gram-negative bacteria), breakdown of gut barrier function, and leakage of bacterial products across the gut lumen. Another potential mechanism could be increased intestinal LPS absorption (i.e., preferential incorporation of LPS into chylomicrons with long-chain dietary fatty acids [42]). Irrespective of the mechanisms leading to higher LPS exposure, the link between LPS and increased inflammation appears convincing [10]. LPS exposure activates the innate immune system by activating the toll-like receptor 4 (TLR4)-MD2 complex, which in turn through the myeloid differentiation primary response protein 88 (MYD88)-dependent and TIR domain-containing adaptor-inducing IFNβ (TRIF) (or MYD88-independent) pathways, induces the production of pro-inflammatory cytokines and interferon (IFN)-inducible proteins, respectively [43]. Thus, the chronic inflammation, oxidative stress, and insulin resistance states of obesity, chronic liver diseases, and, subsequently, HCC may be partly related to endotoxemia. Indeed, there is compelling evidence that obesity might lead to weakening of the gut barrier function and hence greater translocation of LPS [10]. Furthermore, both LPS and flagellin have been shown to induce pro-inflammatory responses in the gut and liver [29, 44–46]. In our study, although circulating anti-LPS or anti-flagellin concentrations increased with BMI, multivariable adjustment for BMI and other perceived confounders (notably alcohol intake, alcohol drinking patterns, and smoking) did not attenuate our observed HCC risk associations with any of the anti-LPS or anti-flagellin biomarkers. Similarly, further adjustment by hsCRP did not appreciably alter our findings — although our single measure of hsCRP may not be sufficient to effectively assess local inflammatory states.

Another interesting observation in our study is a potential sex-specific effect, with a stronger HCC risk observed in men than in women, although the heterogeneity was not statistically significant, likely because of the small number of female HCC cases. Nevertheless, the sex differences observed in our study might be biologically plausible, as men generally have lower innate and adaptive immune responses [47, 48], or because the gut microbiome may alter sex hormone levels and subsequently affect inflammation and autoantibody production, as has been observed in mice [49]. Another animal study has shown that LPS administration resulted in higher levels of LPS-binding protein and pro-inflammatory mediators in male compared to female mice [50]. Interestingly, we previously observed a similar sex-specific effect for endotoxemia associated with the development of colorectal cancer [51]. Future studies with larger numbers of women will be needed to confirm and better understand this potential effect modification by sex.

The main strength of our study is its prospective design, which allowed measurement of the biomarkers in blood samples collected in some cases many years prior to diagnosis. This implies a greater level of confidence in the hypothesis that higher LPS exposure and its various harmful effects are involved in early processes of HCC development. Nevertheless, higher LPS levels also may be indicative of a degree of liver dysfunction, since LPS-binding protein, which binds free circulating LPS, is synthesized in the liver, and individuals with a chronic liver disease may have altered hepatic endotoxin detoxification [52, 53]. Interestingly, the magnitude of our findings was not influenced by additional statistical adjustment for markers of liver dysfunction, further strengthening the argument for an early involvement of endotoxemia in HCC development. Other advantages of the present study are identification of HCC cases based on tumor morphology to ensure the inclusion of only first primary tumors. Research on HCC etiology is hampered by the rarity of the tumor, the inaccurate diagnosis and reporting of primary tumors since the liver is a major site for cancer metastases, and by metabolic changes that occur before cancer diagnosis. Therefore, this prospective study with long and almost complete follow-up, detailed information on dietary/lifestyle factors, and biomarkers measured before cancer diagnosis contributes considerably to our understanding of the role of gut-derived endotoxins in HCC etiology, and possibly HCC risk prediction. In this regard, our study may be criticized for its use of apparently healthy control subjects rather than those with non-HCC chronic liver diseases. But in our opinion, although the latter group may be informative for screening strategies targeted at individuals at higher risk of developing HCC, the former is the most appropriate for both our prospective cohort design and for our primary objective of investigating HCC etiology.

In terms of limitations, our results might be susceptible to confounding, since low LPS and flagellin immunoglobulin levels could indicate better lifestyle and health status. We have attempted to account for this with careful adjustment for relevant lifestyle habits (e.g., alcohol intake, smoking status, and diet), but we cannot discount possible residual confounding. Similarly, we cannot completely rule out the possibility of reverse causality due to the long-term nature of HCC development. We do not have data on incidence of type 2 diabetes and liver diseases (e.g., NAFLD or NASH) or on exposure to aflatoxins, which is uncommon in Western Europe [54]. Infections caused by Gram-negative bacteria of intestinal origin are common among persons with cirrhosis, especially those with more severe liver disease, and in in-hospital settings [55]. However, our study participants were generally healthy at the time of blood collection, and adjustments for biomarkers of liver damage or alcohol intake did not materially change the findings supporting the hypothesis that the presence of endotoxemia might be an additional factor contributing to liver carcinogenesis. In addition, obesity and diabetes, risk factors for HCC, have been shown to be associated with changes in the gut barrier function [56–58], which may contribute to HCC development outside of the spectrum of cirrhosis. However, a possibility that exposure to LPS may trigger the onset of obesity and insulin resistance was also suggested in animal models [59]. Finally, our sample size was relatively small, especially for assessing potential effect modifiers, and liver enzyme levels and HBV/HCV status were only available for 100 cases and 100 controls. Nevertheless, this is the largest prospective study to date on HCC etiology in Western European populations.

## Conclusions

In summary, the findings from this prospective study are in line with the hypothesis that higher exposure to gut-derived endotoxins (due to impaired gut barrier function, lifestyle, or altered gut microbiome) is associated with higher risk of HCC.

## Additional file

Additional file 1:
**Table S1.** Baseline dietary intakes and blood biomarkers of incident HCC cases and matched control subjects within the European Prospective Investigation into Cancer and Nutrition (EPIC) study from 1992 to 2010. **Table S2** Mean (95% CI) anti-LPS and anti-flagellin immunoglobulin levels in controls by sex, age at blood collection, and other baseline characteristics. **Table S3** Incidence rate ratios and 95% confidence intervals of hepatocellular carcinoma according to categories of individual serum anti-LPS and anti-flagellin immunoglobulin levels, EPIC study, 1992–2010. (DOCX 35 kb)

## Abbreviations

EPIC: European Prospective Investigation into Cancer and Nutrition
HBV: Hepatitis B virus
HCC: Hepatocellular carcinoma
HCV: Hepatitis C virus
Ig: Immunoglobulin
LPS: Lipopolysaccharide
NAFLD: Non-alcoholic fatty liver disease
NASH: Non-alcoholic steatohepatitis
