# Supplementary material for: Exposure to bacterial products lipopolysaccharide and flagellin and hepatocellular carcinoma: a nested case-control study
Source: BMC Med. 2017 Apr 4;15:72. doi: 10.1186/s12916-017-0830-8 (PMC5379669; doi:10.1186/s12916-017-0830-8)
Supplement: Additional file 1: — Table S1. Baseline dietary intakes and blood biomarkers of incident HCC cases and matched control subjects within the European Prospective Investigation into Cancer and Nutrition (EPIC) study from 1992 to 2010. Table S2 Mean (95% CI) anti-LPS and anti-flagellin immunoglobulin levels in controls by sex, age at blood collection, and other baseline characteristics. Table S3 Incidence rate ratios and 95% confidence intervals of hepatocellular carcinoma according to categories of individual serum anti-LPS and anti-flagellin immunoglobulin levels, EPIC study, 1992–2010. (DOCX 35 kb) [file 12916_2017_830_MOESM1_ESM.docx]

**Additional file 1**

| **Table S1.** Baseline dietary intakes and blood biomarkers of incident HCC cases and matched control subjects within the European Prospective Investigation into Cancer and Nutrition (EPIC) study from 1992 to 2010*. | | | | | | | | |  |  |
| --- | --- | --- | --- | --- | --- | --- | --- | --- | --- | --- |
| Characteristic | | | | | Case subjects (N = 139) | | Matched control subjects (N = 139)^†^ | | | |
| Daily intake, mean (SD) | | | | |  | |  | |  |  |
|  | | | Energy (kcal) | | 2265.7 (1021.2) | | 2270 (626) | |  |  |
|  | | | Alcohol (g) | | 22.2 (34.6) | | 15.2 (19.4) | |  |  |
|  | | | Total dietary fiber (g) | | 21.0 (8.9) | | 23.2 (7.8) | |  |  |
|  | | | Red and processed meat (g) | | 82.0 (53.0) | | 88.9 (57.0) | |  |  |
|  | | | Fruits and vegetables (g) | | 412.3 (271.7) | | 460.7 (286.6) | |  |  |
|  | | | Coffee (g) | | 309.4 (358.1) | | 379.7 (422.9) | |  |  |
| Baseline blood biomarkers, mean (SD) | | | | |  | |  | |  |  |
|  | | | Anti-LPS IgG | | 1.9 (0.72) | | 1.68 (0.68) | | | |
|  | | | Anti-LPS IgA | | 2.37 (0.83) | | 1.96 (0.76) | | | |
|  | | | Anti-Flagellin IgG | | 1.72 (0.69) | | 1.51 (0.63) | | | |
|  | | | Anti-Flagellin IgA | | 2.09 (0.81) | | 1.72 (0.71) | | | |
|  | | | Alanine aminotransferase (U/L)^§^ | | 34.1 (2.2) | | 18.0 (1.6) | | | |
|  | | | Aspartate aminotransferase (U/L)^§^ | | 39.6 (2.0) | | 19.1 (1.3) | | | |
|  | | | Gamma glutamyl transferase (U/L)^§^ | | 80.6 (3.3) | | 24.0 (1.8) | | | |
|  | | | Liver-specific alkaline phosphatase (U/L)^§^ | | 83.1 (1.5) | | 60.3 (1.3) | | | |
|  | | | Albumin (g/L)^§^ | | 3.9 (1.1) | | 4.2 (1.1) | | | |
|  | | | Total bilirubin (mg/dL)^§^ | | 0.6 (2.0) | | 0.4 (1.6) | | | |
| § Geometric means and SDs, available for 100 cases and 100 controls. | | | | | | | | |  |  |

**Table S2.** Mean (95% CI) anti-LPS and anti-Flagellin immunoglobulin levels^#^ in controls by sex, age at blood collection, and other baseline characteristics.

| **Characteristic** | | **Anti-LPS IgG & IgA** | | **95% CI** | | **Anti-Flagellin IgG & IgA** | | **95% CI** | | | **Anti-LPS IgG + Anti-Flagellin IgG** | | **95% CI** | | | **Anti-LPS IgA + Anti-Flagellin IgA** | | **95% CI** | | | **Anti-LPS IgG & IgA + Anti-Flagellin IgG & IgA** | | **95% CI** | | |  |  |
| --- | --- | --- | --- | --- | --- | --- | --- | --- | --- | --- | --- | --- | --- | --- | --- | --- | --- | --- | --- | --- | --- | --- | --- | --- | --- | --- | --- |
| **Baseline coffee intake, cup/day** | | | |  | |  | |  | |  |  | |  | |  |  | |  | |  |  | |  | |  |  | |
| Non-drinker | | 3.37 | | 2.65 | | 4.09 | | 2.86 | | 2.22 | 3.51 | | 2.90 | | 2.25 | 3.55 | | 3.34 | | 2.57 | 4.11 | | 6.24 | | 4.95 | 7.52 | |
| <1 | | 3.84 | | 3.51 | | 4.17 | | 3.30 | | 3.00 | 3.59 | | 3.33 | | 3.03 | 3.63 | | 3.81 | | 3.46 | 4.16 | | 7.14 | | 6.55 | 7.73 | |
| 1-2 | | 3.85 | | 3.23 | | 4.46 | | 3.29 | | 2.74 | 3.84 | | 3.36 | | 2.81 | 3.91 | | 3.78 | | 3.12 | 4.43 | | 7.14 | | 6.04 | 8.23 | |
| ≥ 2 | | 3.36 | | 2.98 | | 3.74 | | 3.20 | | 2.85 | 3.54 | | 3.02 | | 2.67 | 3.36 | | 3.54 | | 3.13 | 3.95 | | 6.56 | | 5.87 | 7.24 | |
| *P-value** | | *0.983* | |  | |  | | *0.392* | |  |  | | *0.738* | |  |  | | *0.679* | |  |  | | *0.677* | |  |  | |
| **Baseline dietary fiber intake, g/day** | | | |  | |  | |  | |  |  | |  | |  |  | |  | |  |  | |  | |  |  | |
| <14.3 | | 3.91 | | 3.47 | | 4.36 | | 3.40 | | 3.00 | 3.79 | | 3.33 | | 2.93 | 3.74 | | 3.98 | | 3.50 | 4.45 | | 7.31 | | 6.51 | 8.11 | |
| 14.3-19.53 | | 3.58 | | 3.14 | | 4.01 | | 3.07 | | 2.68 | 3.45 | | 3.21 | | 2.82 | 3.61 | | 3.43 | | 2.97 | 3.89 | | 6.64 | | 5.87 | 7.42 | |
| 19.54-24.98 | | 3.53 | | 3.09 | | 3.98 | | 3.21 | | 2.82 | 3.60 | | 3.10 | | 2.70 | 3.50 | | 3.65 | | 3.18 | 4.11 | | 6.75 | | 5.96 | 7.53 | |
| >24.98 | | 3.54 | | 3.09 | | 3.99 | | 3.23 | | 2.83 | 3.62 | | 3.11 | | 2.71 | 3.52 | | 3.65 | | 3.18 | 4.13 | | 6.77 | | 5.97 | 7.57 | |
| *P-value** | | *0.253* | |  | |  | | *0.683* | |  |  | | *0.397* | |  |  | | *0.483* | |  |  | | *0.398* | |  |  | |
| **Smoking status**^†^ | |  | |  | |  | |  | |  |  | |  | |  |  | |  | |  |  | |  | |  |  | |
| Never | | 3.70 | | 3.36 | | 4.04 | | 3.15 | | 2.85 | 3.44 | | 3.28 | | 2.98 | 3.59 | | 3.57 | | 3.21 | 3.92 | | 6.85 | | 6.25 | 7.45 | |
| Former | | 3.66 | | 3.29 | | 4.03 | | 3.43 | | 3.10 | 3.75 | | 3.19 | | 2.85 | 3.52 | | 3.90 | | 3.50 | 4.29 | | 7.08 | | 6.42 | 7.74 | |
| Smoker | | 3.41 | | 2.92 | | 3.91 | | 3.05 | | 2.61 | 3.49 | | 2.99 | | 2.54 | 3.43 | | 3.47 | | 2.95 | 4.00 | | 6.46 | | 5.58 | 7.34 | |
| *P-value** | | *0.622* | |  | |  | | *0.308* | |  |  | | *0.565* | |  |  | | *0.339* | |  |  | | *0.538* | |  |  | |
| **Baseline alcohol intake, g/d** | | | |  | |  | |  | |  |  | |  | |  |  | |  | |  |  | |  | |  |  | |
| Non drinker | | 3.46 | | 2.82 | | 4.10 | | 2.91 | | 2.35 | 3.48 | | 2.93 | | 2.36 | 3.50 | | 3.44 | | 2.77 | 4.11 | | 6.37 | | 5.23 | 7.51 | |
| >0-6(M)/>0-3(W) | | 3.57 | | 3.15 | | 3.99 | | 3.12 | | 2.75 | 3.50 | | 3.27 | | 2.89 | 3.65 | | 3.42 | | 2.98 | 3.87 | | 6.70 | | 5.94 | 7.45 | |
| >6-12(M)/>3-12(W) | | 3.78 | | 3.28 | | 4.28 | | 3.28 | | 2.84 | 3.72 | | 3.18 | | 2.73 | 3.62 | | 3.88 | | 3.36 | 4.41 | | 7.06 | | 6.17 | 7.95 | |
| >12-24 | | 3.76 | | 3.24 | | 4.28 | | 3.44 | | 2.98 | 3.89 | | 3.45 | | 2.98 | 3.91 | | 3.75 | | 3.21 | 4.30 | | 7.20 | | 6.27 | 8.12 | |
| >24-60 | | 3.61 | | 3.08 | | 4.14 | | 3.30 | | 2.83 | 3.76 | | 2.97 | | 2.50 | 3.44 | | 3.93 | | 3.38 | 4.49 | | 6.90 | | 5.97 | 7.84 | |
| >60-96(M)/>60(W) | | 3.57 | | 2.24 | | 4.89 | | 3.22 | | 2.05 | 4.38 | | 3.30 | | 2.12 | 4.48 | | 3.48 | | 2.09 | 4.87 | | 6.78 | | 4.44 | 9.13 | |
| *P-value** | | *0.744* | |  | |  | | *0.501* | |  |  | | *0.462* | |  |  | | *0.802* | |  |  | | *0.604* | |  |  | |
| **Lifetime alcohol drinking pattern** | | | |  | |  | |  | |  |  | |  | |  |  | |  | |  |  | |  | |  |  | |
| Never drinkers | | 3.79 | | 2.97 | | 4.60 | | 2.86 | | 2.15 | 3.57 | | 3.02 | | 2.29 | 3.76 | | 3.62 | | 2.78 | 4.47 | | 6.64 | | 5.20 | 8.09 | |
| Light drinkers | | 3.58 | | 2.91 | | 4.26 | | 3.00 | | 2.41 | 3.59 | | 3.35 | | 2.74 | 3.96 | | 3.23 | | 2.53 | 3.93 | | 6.58 | | 5.38 | 7.78 | |
| Never heavy drinkers | | 3.66 | | 3.34 | | 3.98 | | 3.33 | | 3.05 | 3.61 | | 3.22 | | 2.93 | 3.51 | | 3.77 | | 3.44 | 4.10 | | 6.99 | | 6.42 | 7.56 | |
| Always heavy drinkers | | 3.96 | | 1.26 | | 6.66 | | 3.18 | | 0.82 | 5.55 | | 3.89 | | 1.45 | 6.32 | | 3.26 | | 0.46 | 6.05 | | 7.14 | | 2.34 | 11.95 | |
| Periodically heavy drinkers | | 3.77 | | 3.14 | | 4.41 | | 3.31 | | 2.75 | 3.86 | | 3.35 | | 2.78 | 3.92 | | 3.73 | | 3.07 | 4.39 | | 7.08 | | 5.95 | 8.21 | |
| Former heavy drinkers | | 4.12 | | 1.42 | | 6.82 | | 3.28 | | 0.91 | 5.64 | | 2.28 | | 0.16 | 4.71 | | 5.11 | | 2.32 | 7.91 | | 7.39 | | 2.59 | 12.19 | |
| Former light drinkers | | 2.84 | | 0.93 | | 4.75 | | 2.75 | | 1.08 | 4.42 | | 2.64 | | 0.92 | 4.36 | | 2.95 | | 0.98 | 4.93 | | 5.59 | | 2.20 | 8.99 | |
| *P-value** | | *0.979* | |  | |  | | *0.860* | |  |  | | *0.919* | |  |  | | *0.728* | |  |  | | *0.974* | |  |  | |
| **Diabetes at baseline** | |  | |  | |  | |  | |  |  | |  | |  |  | |  | |  |  | |  | |  |  | |
| No | | 3.68 | | 3.43 | | 3.93 | | 3.20 | | 2.98 | 3.42 | | 3.19 | | 2.97 | 3.42 | | 3.68 | | 3.42 | 3.95 | | 6.88 | | 6.43 | 7.32 | |
| Yes | | 3.96 | | 3.11 | | 4.81 | | 3.47 | | 2.73 | 4.22 | | 3.21 | | 2.44 | 3.98 | | 4.22 | | 3.33 | 5.11 | | 7.43 | | 5.91 | 8.95 | |
| *P-value** | | *0.528* | |  | |  | | *0.493* | |  |  | | *0.968* | |  |  | | *0.254* | |  |  | | *0.490* | |  |  | |

* All P-values are based on a test of linear trend, except P-values for heterogeneity by country, geographical region, sex, smoking status and educational level.

^†^ Number of missing/unknown among controls: smoking = 10.

**Table S3.** Incidence rate ratios and 95% confidence intervals of hepatocellular carcinoma according to categories of individual serum anti-LPS and anti-Flagellin immunoglobulin levels^#^, EPIC study, 1992-2010.

| **Biomarker** | | **IRR (95% CI)** | | | |  | **OR _per ↑1 unit_** |
| --- | --- | --- | --- | --- | --- | --- | --- |
|  |  | **Q1** | **Q2** | **Q3** | **Q4** | ***P* _trend_** |  |
| Anti-LPS IgG, n ca/co | | 33/34 | 19/35 | 42/35 | 45/35 |  |  |
|  | Matching factors† | ref. | 0.58(0.28-1.21) | 1.67(0.8-3.48) | 3.00(1.1-8.23) | 0.042 | 2.85(1.62-5.03) |
|  | Multivariable ‡ | ref. | 0.60(0.23-1.58) | 1.94(0.73-5.15) | 4.13(1.06-16.07) | 0.040 | 3.94(1.80-8.63) |
|  | Adjusted for dietary factors§ | ref. | 0.64(0.23-1.80) | 2.42(0.85-6.90) | 5.40(1.3-22.42) | 0.019 | 4.67(2.03-10.76) |
|  | Adjusted for Fischer’s ratio* | ref. | 0.33(0.09-1.23) | 1.72(0.50-5.88) | 1.79(0.26-12.25) | 0.239 | 2.68(1.07-6.72) |
| Anti-LPS IgA, n ca/co | | 18/34 | 23/35 | 35/35 | 63/35 |  |  |
|  | Matching factors† | ref. | 1.62(0.69-3.8) | 2.45(1.06-5.64) | 7.43(2.77-19.88) | <.0001 | 3.11(1.93-5.01) |
|  | Multivariable ‡ | ref. | 2.01(0.65-6.25) | 3.15(1.07-9.28) | 8.67(2.27-33.16) | 0.001 | 3.76(1.91-7.37) |
|  | Adjusted for dietary factors§ | ref. | 2.44(0.74-8.09) | 3.67(1.18-11.43 | 10.49(2.50-44.00) | 0.001 | 4.01(1.96-8.21) |
|  | Adjusted for Fischer’s ratio* | ref. | 1.12(0.29-4.29) | 3.63(1.01-13.11 | 5.18(0.98-27.50) | 0.011 | 2.92(1.38-6.17) |
| Anti-Flagellin IgG, n ca/co | | 26/34 | 36/36 | 29/34 | 48/35 |  |  |
|  | Matching factors† | ref. | 1.39(0.69-2.8) | 1.42(0.6-3.35) | 3.08(1.17-8.10) | 0.032 | 2.9(1.57-5.35) |
|  | Multivariable ‡ | ref. | 1.76(0.69-4.49) | 2.11(0.69-6.45) | 5.83(1.64-20.72) | 0.008 | 3.67(1.72-7.83) |
|  | Adjusted for dietary factors§ | ref. | 1.81(0.69-4.75) | 2.93(0.86-10) | 5.72(1.55-21.08) | 0.008 | 3.7(1.69-8.11) |
|  | Adjusted for Fischer’s ratio * | ref. | 1.79(0.61-5.27) | 3.59(0.9-14.36) | 2.81(0.59-13.49) | 0.105 | 2.87(1.16-7.13) |
| Anti-Flagellin IgA, n ca/co | | 18/35 | 26/34 | 36/35 | 59/35 |  |  |
|  | Matching factors† | ref. | 1.69(0.75-3.83) | 2.77(1.17-6.58) | 6.29(2.44-16.22) | <.0001 | 2.82(1.77-4.49) |
|  | Multivariable ‡ | ref. | 3.49(1.14-10.64) | 4.73(1.35-16.52) | 7.05(2.07-24.01) | 0.003 | 2.78(1.54-5.04) |
|  | Adjusted for dietary factors§ | ref. | 3.85(1.24-12.01) | 5.03(1.37-18.47) | 6.08(1.78-20.74) | 0.007 | 2.63(1.45-4.78) |
|  | Adjusted for Fischer’s ratio * | ref. | 2.44(0.69-8.61) | 4.06(0.95-17.31) | 3.23(0.82-12.69) | 0.094 | 1.92(0.98-3.77) |
| † IRRs and 95% confidence intervals were estimated by conditional logistic regression conditioned on the matching factors. | | | | | | | |
| ‡ Base model further adjusted for smoking status (never, former, current), body mass index (continuous), baseline alcohol intake (continuous), coffee intake (continuous), lifetime alcohol drinking pattern (always heavy, periodically heavy, former heavy, never heavy, former light, light, and never drinkers), physical activity (active, moderately active, moderately inactive, inactive), and level of education (none, primary school, secondary school, more than secondary school, not specified). | | | | | | | |
| § Multivariable model + baseline dietary fiber (g/d), fish and seafood products (g/d), and total energy (kcal/d). | | | | | | | |
| * Multivariable model + Fischer’s ratio calculated as the molar ratio of branched-chain amino acids (leucine, valine, isoleucine) to aromatic amino acids (phenylalanine, tyrosine, histidine and tryptophan), an indicator of hepatic functional reserve and the severity of liver dysfunction. | | | | | | | |
